# Imeglimin Attenuates Skeletal Muscle Atrophy in Mouse Models of Obesity and Ageing

**DOI:** 10.1002/jcsm.70354

**Published:** 2026-07-28

**Authors:** Yangli Ye, Diandian Qian, Ikumi Nomura, Naoki Kobayashi, Yukiko Shimizu, Tadashi Okamura, Motoharu Awazawa, Kohjiro Ueki

**Affiliations:** ^1^ Department of Geriatrics Zhongshan Hospital, Fudan University Shanghai China; ^2^ Department of Molecular Diabetology, Diabetes Research Center Japan Institute for Health Security Tokyo Japan; ^3^ Department of Laboratory Animal Medicine Japan Institute for Health Security Tokyo Japan; ^4^ Department of Molecular Diabetology, Graduate School of Medicine The University of Tokyo Tokyo Japan

**Keywords:** ageing, imeglimin, muscle atrophy, obesity, sarcopenia

## Abstract

**Background:**

Sarcopenia is a major contributor to frailty and mortality in ageing and obesity and is tightly linked to metabolic dysfunction. Imeglimin is a first‐in‐class oral hypoglycaemic agent targeting mitochondrial function; however, despite the central role of mitochondria in skeletal muscle homeostasis, its effects on skeletal muscle under sarcopenia‐relevant conditions remain unclear.

**Methods:**

Imeglimin was administered to male C57BL/6 mice with high‐fat diet (HFD)–induced obesity for 6 weeks and to naturally aged (18 months old) male mice for 12 weeks. Skeletal muscle fibre morphology and transcriptomic profiles were analysed in fast‐ and slow‐twitch muscles. In parallel, C2C12 myotubes were exposed to palmitate with or without imeglimin, and inflammatory gene expression and reactive oxygen species (ROS) generation were assessed.

**Results:**

Imeglimin significantly increased the cross‐sectional area (CSA) of Type II fibres in the extensor digitorum longus (EDL) muscle of HFD‐fed mice (+66%, *p* < 0.01 vs. controls). Transcriptomic analyses revealed suppression of conserved molecular signatures of muscle atrophy, including activation of immediate‐early genes and inflammatory pathways (−62% to −79%, *p* < 0.05 vs. HFD‐fed mice). In palmitate‐treated C2C12 myotubes, imeglimin attenuated lipotoxicity‐induced inflammatory gene expression (−28% to −72%, *p* < 0.05 vs. controls) with reduced ROS generation, consistent with its cell‐autonomous effect on myocytes. Notably, in naturally aged mice, 12‐week imeglimin treatment preserved EDL muscle fibre size (+14%, *p* < 0.05 vs. controls) without altering systemic glucose tolerance, accompanied by transcriptomic changes overlapping with those observed in the HFD model (−27% to −82%, *p* < 0.05 vs. aged controls).

**Conclusions:**

Imeglimin attenuates skeletal muscle atrophy in obesity and ageing, accompanied by coordinated suppression of stress‐ and inflammation‐associated transcriptional programmes. These findings indicate that pharmacological regulation of mitochondrial stress responses influences skeletal muscle vulnerability under chronic metabolic stress and identify skeletal muscle as a previously underappreciated target of imeglimin action.

## 1 Introduction

Sarcopenia is a prevalent and growing concern in many developed countries, affecting not only ageing populations but also individuals with obesity and diabetes mellitus [1, 2]. In these settings, loss of skeletal muscle mass and metabolic dysfunction interact bidirectionally, thereby exacerbating functional decline and systemic pathology. Despite extensive efforts to elucidate the molecular mechanisms underlying sarcopenia, effective pharmacological strategies for preserving skeletal muscle mass remain limited. In parallel, the expanding use of pharmacological interventions for metabolic diseases has, with its potent weight‐reducing effects, heightened interest in understanding how chronic metabolic stress and its treatment influence skeletal muscle integrity [3].

Imeglimin is a recently developed, first‐in‐class metabolic modulator targeting mitochondrial function and is currently approved as an oral hypoglycaemic agent in Japan. It was designed to improve mitochondrial bioenergetics and to reduce reactive oxygen species (ROS)–associated cellular stress across multiple metabolic tissues [4]. Consistent with this mechanism, previous studies have established its glucose‐lowering effects [5, 6, 7] and have reported pleiotropic metabolic actions, including amelioration of hepatic steatosis [8] and improvement of obesity‐associated dysbiosis in mice [9]. Given the central role of mitochondrial function in skeletal muscle physiology and vulnerability to atrophic stress, it is plausible that imeglimin may influence skeletal muscle homeostasis. However, the impact of imeglimin on skeletal muscle, particularly under conditions relevant to sarcopenia, remains largely unexplored.

Here, using mouse models of obesity and ageing, we demonstrate that imeglimin attenuates skeletal muscle atrophy under chronic metabolic stress. In a high‐fat diet (HFD)–induced model of obesity, imeglimin significantly preserved muscle fibre size in fast‐twitch muscle enriched in Type IIX/D fibres, which are particularly susceptible to atrophic stimuli. Transcriptomic analyses revealed suppression of HFD‐associated activation of immediate‐early genes and inflammatory pathways, molecular features commonly observed in ageing‐related sarcopenia [10]. Consistent with these findings, imeglimin treatment also preserved skeletal muscle fibre size in naturally aged male mice following 12 weeks of administration. Exploratory analyses also suggested potential effects of imeglimin on bone‐related parameters in aged mice. Together, these findings identify skeletal muscle as a metabolically responsive target of imeglimin and provide preclinical evidence linking modulation of metabolic stress‐responsive pathways to preservation of musculoskeletal integrity in obesity and ageing.

## 2 Materials and Methods

### 2.1 Animals

The obese mouse model was established as previously described [9]. Briefly, male C57BL/6J‐DIO mice (fed with D12492, 60% kcal from fat; Research Diets Inc) and the control age‐matched male C57BL/6 wild‐type mice (The Jackson Laboratory) were delivered at 18 weeks of age and thereafter continued to be fed with a standard rodent normal chow diet (NCD) (CE‐2, Japan CLEA; Harlan), HFD (D12492, 60% kcal from fat; Research Diets Inc) or HFD mixed with imeglimin hydrochloride (0.6%, Sumitomo Pharma; estimated 300 mg kg^−1^ day^−1^) for 6 weeks. For the ageing experiments, 76‐week‐old male C57BL/6J mice were fed standard rodent diet (D11112201, Research Diet Inc) with or without imeglimin hydrochloride (0.3%; estimated 400 mg kg^−1^ day^−1^) for 12 weeks. Young control mice were fed standard diet from 4 to 12 weeks of age. Grip strength was measured in an additional cohort of male C57BL/6J mice at 90 weeks of age after 7 weeks of imeglimin treatment using a Grip Strength Meter (MK‐380V, Muromachi Kikai). Each mouse was tested five times, and the average value was used for analyses. The mice were housed under specific pathogen‐free conditions with a 12–12‐h light–dark cycle at 22°C. All animals were randomly assigned to the experimental groups, and the tests were performed blinded. Sample sizes for animal experiments were calculated based on power calculations with an alpha of 0.8. All animal experiments were approved by the President of the Japan Institute for Health Security (JIHS) under the institutional guidelines and national laws.

### 2.2 Histological Analyses

The skeletal muscle tissues were fixed in 4% paraformaldehyde and then paraffin embedded. After antigen retrieval and blocking, slides were incubated with the primary antibodies against MYH1 Antibody (M‐4276, SIGMA, Type IIX/D fast‐twitch fibres) and MYH7 (ab234431, Abcam, Type I slow‐twitch fibres). The secondary antibodies were anti‐mouse secondary antibody (K4001, DAKO) and anti‐rabbit secondary antibody (K4003, DAKO), respectively. Immunostaining was visualized by DAB. The slides were observed using a BZ‐X700 (Keyence), and cross‐sectional areas (CSAs) of myofibres were analysed using ImageJ from five randomly selected images per sample.

### 2.3 RNA Sequencing Analysis

Total RNA was isolated from the EDL using TRIZOL reagent (Invitrogen), followed by column purification with FavorPrep Tissue Total RNA Mini Kit (FAVORGEN). RNA sequencing was performed using NovaSeq6000 system (Illumina). Raw sequencing reads were aligned to a mouse reference genome (GRCm39) using STAR, and gene expression was quantified using RSEM. The raw read counts were normalized with transcripts per million (TPM). Differential gene expression, heatmap visualization and *k*‐means clustering were conducted using the R programming language (version 4.x.x) with the following packages: *readxl* (for data import), *dplyr*, *tidyr*, *tibble* (for data manipulation), cluster (for clustering) and pheatmap (for heat map generation). The gene ontology (GO) analyses were performed using DAVID Functional Annotation Tools by focusing on KEGG pathways.

### 2.4 Quantitative Real‐Time PCR

Total RNA was isolated by TRIZOL reagent (Invitrogen) and reverse‐transcribed into cDNA. Quantitative real‐time PCR analyses were performed using the StepOnePlus Real‐Time PCR System (Applied Biosystems). The relative gene expression was normalized to internal control gene expression using the comparative Ct method in the same sample. All primers and probes were purchased from Applied Biosystems.

### 2.5 Immunofluorescence Staining of Plasminogen Activator Inhibitor‐1 (PAI‐1/Srepine1)

Paraffin‐embedded tissue sections were deparaffinized, rehydrated through graded ethanol solutions, subjected to antigen retrieval, blocked and incubated with anti‐PAI‐1 antibody (NBP1‐19773, lot C‐4, Novus Biologicals) diluted 1:25 in antibody diluent overnight at 4°C. Alexa Fluor 555‐conjugated secondary antibody was used for detection. Nuclei were counterstained with DAPI, and fluorescence images were obtained using a fluorescence microscope.

### 2.6 Cells and Cell Culture

Palmitate (Sigma‐Aldrich) was dissolved in 100% ethanol (200‐mM stock) and conjugated with 20% BSA (fatty acid‐free, Sigma‐Aldrich) to a final concentration of 10‐mM palmitate. C2C12 myoblasts (ATCC) were differentiated into myotubes with Dulbecco's Modified Eagle Medium (DMEM) containing 2% horse serum for 5–6 days. Differentiated C2C12 myotubes predominantly exhibit fast‐twitch/Type II muscle characteristics, including MYH1‐associated myosin heavy chain expression, consistent with the glycolytic characteristics in EDL muscle in vivo. The primary muscle interstitial cells were isolated from hindlimb skeletal muscles (quadriceps and gastrocnemius) of 20‐week‐old C57BL/6 male mice by enzymatic digestion and cultured in DMEM Low Glucose supplemented with 20% FBS. Cells were incubated at 37°C and 5% CO_2_. After 24 h, the medium was replaced to remove nonadherent cells, and the resulting adherent cells were expanded and subjected to the experiments after two passages. Palmitate of 0.5‐mM concentration or control BSA solution was administered with or without imeglimin of 2.5 mM. Imeglimin was added to the medium 10 min prior to palmitate. All the cell culture experiments were conducted without blinding.

### 2.7 ROS Assay

Cellular ROS levels were assessed using DCFH‐DA reagent (ROS Assay Kit, Dojindo, Japan). Differentiated C2C12 myotubes were incubated with palmitate of 0.5‐mM concentration or control BSA solution with or without 2.5 mM of imeglimin for 24 h followed by DCFH‐DA fluorescence imaging.

### 2.8 Immunoblotting

The cells or tissues were lysed in Nonidet‐P 4‐containing lysis buffer supplemented with protease inhibitors. The proteins were separated by SDS‐PAGE and transferred onto PVDF membrane. The membrane was incubated with the primary antibodies, followed by incubation with HRP‐conjugated secondary antibodies and ECL detection. The antibodies against phosphorylated Akt (Ser473: #4060), total Akt (#4691), phosphorylated‐JNK (#4668), JNK (#9252), phosphorylated‐p38 (#4511), p38 (#8690), phosphorylated‐IκBα (#2859), IκBα (#9242), phosphorylated‐mTOR (#2971), mTOR (#2983) and Cox IV (#4844) were purchased from Cell Signalling Technology. Calnexin antibody (#10427‐2‐AP) was purchased from Proteintech. Total OXPHOS Rodent WB Antibody Cocktail (#ab110413) was purchased from Abcam. Antibodies were used at a 1:1000 dilution. To avoid aggregation of mitochondrial membrane proteins, samples for OXPHOS western blotting were heated at 50°C for 10 min instead of boiling. For the total protein immunoblot, the membrane used for the phosphorylated protein was reprobed for the total protein. The secondary antibody (A6154, Santa‐Cruz) was used at a 1:3000 dilution. For the quantitative analyses, the densitometry was performed with the Fiji software.

### 2.9 Metabolic Assays

After 11 weeks of treatment with imeglimin, the insulin tolerance test (i.p. insulin 1.5 i.U./kg after 3 h of starvation) and the glucose tolerance test (i.p. 2 g/kg body weight of glucose after fasting for 6 h) were performed. Blood glucose concentrations were measured from the tail vein blood using Glutest Mint (Sanwa Kagaku Kenkyusho).

### 2.10 ELISA Assays

Plasma concentrations of insulin (Morinaga Bioscience), PINP (SEA957Mu, CLOUD‐CLONE CORP, WUHAN) and CTX‐1 (ABIN6955118, ANTIBODIES ONLINE) were measured by ELISA according to the manufacturers' protocol.

### 2.11 microCT Analysis

Femoral bone microstructure was assessed using a microcomputed tomography system (Cosmo Scan FX, Rigaku Corporation, Japan). Scanning parameters: 90 kV, 88 μA, 20‐μm voxel size and 2‐min scan. Projection images were reconstructed using BMD Analysis software (Rigaku Corporation). The region of interest was defined 1 mm proximal to the distal growth plate over a 0.7‐mm segment [11]. Morphometric parameters were quantified, including tissue volume (TV, mm^3^), bone volume (BV, mm^3^), bone surface (BS, mm^2^), BS/TV (1/mm) and BV/TV (%). BMD was calibrated using a hydroxyapatite phantom (MHA‐287φ4.5, QRM, Germany) and calculated for trabecular and cortical bone.

### 2.12 Statistical Analysis

Data were presented as mean ± SEM. Statistical analyses were performed using one‐way ANOVA with the Holm–Sidak multiple comparison test unless otherwise specified. The normality of distribution was calculated using the D'Agostino and Pearson omnibus normality test where applicable. For the calculations of the equality of variance in one‐way ANOVA, Brown–Forsythe test was used. Outliers were identified by ROUT method (Q = 5%) and excluded from the analyses. All the analyses were performed with GraphPad PRISM Version 10.0.3.

## 3 Results

### 3.1 Imeglimin Protected Mice From HFD‐Associated Muscle Atrophy of EDL

**FIGURE 1:**
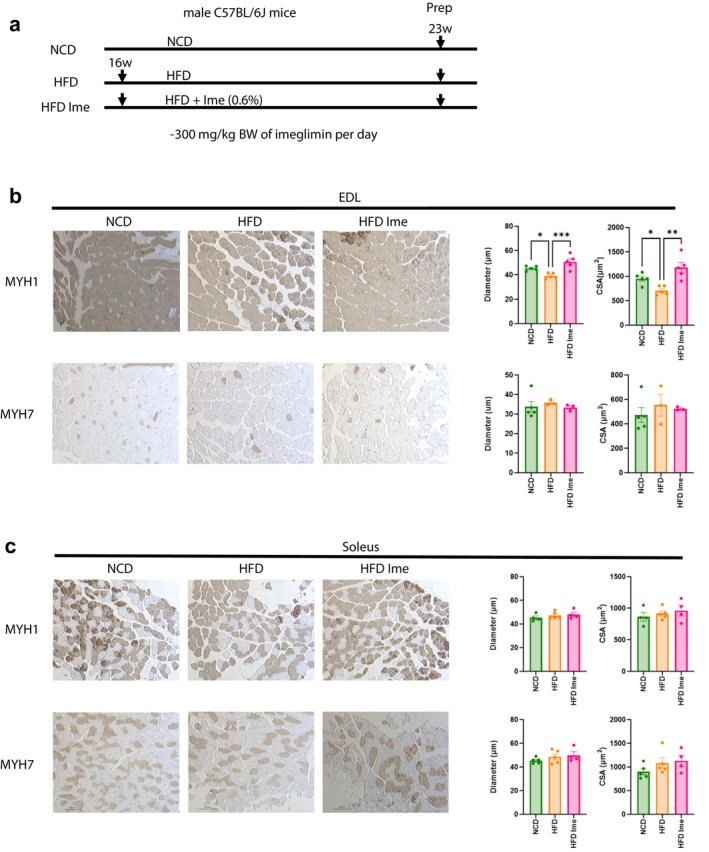
Histology of EDL and soleus muscles in the HFD‐fed mice with imeglimin administration. (a) A scheme showing the experimental setting. (b, c) The histological analyses of (b) EDL and (c) soleus muscles of mice fed with chow diet (NCD), high‐fat diet (HFD) or HFD with imeglimin (HFD Ime). MYH1: Type IIX/D fast‐twitch muscle fibre staining; MYH7: Type I slow‐twitch muscle fibre staining. The left panels show representative images, and the right panels show the quantifications of cross‐sectional area (CSA) and diameter of each fibre type (*n* = 5). *p < 0.05, **p < 0.01, ***p < 0.001. The graphs represent mean ± SEM.

In a previous study, we demonstrated that administering imeglimin for 6 weeks to 18‐week‐old HFD‐fed obese mice (Figure 1a) significantly enhanced energy expenditure, protected mice from weight gain and ameliorated HFD‐induced dysbiosis in the gut [9]. Given that sarcopenia is another significant comorbidity associated with obesity [1], we used this same mouse model to investigate the effects of imeglimin on skeletal muscles, specifically its potential to prevent sarcopenia.

We first subjected hindlimb skeletal muscle strips to histological assessments. Staining of the EDL muscle with a MYH1 antibody revealed that both the diameter and CSA of Type IIX/D fibres, the dominant fibre type in the EDL, were significantly lower in HFD‐fed mice compared to lean controls fed an NCD. This finding suggests that the HFD in our model led to muscle atrophy in Type IIX/D fast‐twitch fibres. Notably, the CSA of Type IIX/D fibres in the EDL of imeglimin‐administrated HFD‐fed mice was significantly larger than that of HFD‐fed mice, reaching levels comparable to those of the lean control mice (Figure 1b, **upper panels**). Conversely, the diameter and CSA of Type I fibres (stained with MYH7) in the EDL remained unchanged (Figure 1b, **lower panels**), as did those of both Type I and Type IIX/D fibres in the soleus muscles (Figure 1c). These morphological assessments indicated that imeglimin administration prevented HFD‐associated muscle atrophy predominantly in Type IIX/D fast‐twitch EDL muscle.

### 3.2 Imeglimin Ameliorated HFD‐Associated Gene Expression Changes in EDL

**FIGURE 2:**
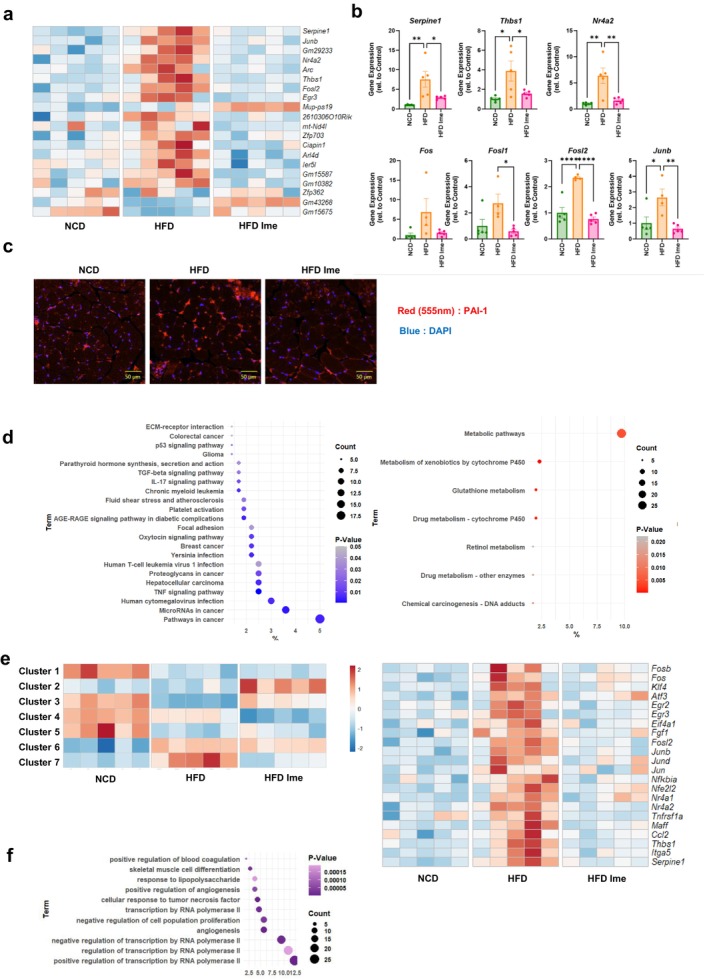
Transcriptome of EDL in HFD‐fed mice with imeglimin administration. (a) The heatmap showing the gene expressions of top 20 differentially expressed genes (DEG) in the transcriptome analysis of EDL from the mice fed with NCD, HFD or HFD + imeglimin (HFD Ime). (b) Real‐time PCR analyses of indicated gene expressions in EDL of mice (*n* = 5). (c) Representative immunofluorescence staining of PAI‐1 (Serpine1) in EDL muscles from NCD, HFD or HFD Ime. The right panel shows the quantification of PAI‐1 fluorescence intensity. Scale bar = 50 μm. (d) The results of gene ontology analyses of the downregulated genes (left panel) and upregulated genes (right panel) in the EDL of HFD Ime group compared to the HFD group. (e) The results of *k*‐means clustering of EDL transcriptome (left panel) and the heatmap showing the expressions of genes included in Cluster 7 (right panel). (f) The results of gene ontology analyses of genes in Cluster 7 of *k*‐means clustering analyses. *p < 0.05, **p < 0.01 and ***p < 0.001. The graphs represent mean ± SEM.

To further characterize the imeglimin‐induced changes in EDL, we performed transcriptome analyses. The top 20 differentially expressed genes (DEG) among the three cohorts of mice showed a prominent enrichment of immediate‐early genes (IEGs) such as *Junb*, *Fosl2* and *Egr3* as well as TGF‐β pathway‐related genes such as *Serpine1* and *Thbs1*, together with an inflammatory IEG *Nr4a2*. These genes were all upregulated in the EDL of HFD‐fed mice, and imeglimin administration suppressed their expression to the levels of NCD‐fed controls (Figure 2a). Real‐time PCR analyses further confirmed these mRNA expression changes in EDL (Figure 2b). Immunofluorescence staining in EDL muscles revealed that HFD feeding markedly increased PAI‐1 (encoded by *Serpine1*) immunoreactivity, whereas imeglimin administration substantially reduced the elevated PAI‐1 signals (Figure 2c), consistent with the transcriptomic and qPCR analyses. GO analyses of the genes downregulated by imeglimin (nominal *p* < 0.05) in the EDL of HFD‐fed animals confirmed the enrichment of TGF‐β signalling pathway and also suggested the downregulation of TNF signalling (Figure 2d, **left panel**). On the other hand, GO analyses of the upregulated genes (nominal *p* < 0.05) in the EDL of imeglimin‐administered HFD‐fed animals compared to the HFD‐fed controls showed an enrichment of glutathione metabolism‐related genes (Figure 2d, **right panel**), suggesting potential antioxidant pathway activation by imeglimin. An independent analysis of EDL transcriptomes using the *k*‐means clustering method identified seven distinct gene clusters, where Cluster 2 contained the genes specifically upregulated in imeglimin‐administered mice, whereas Cluster 7 represented the genes upregulated in HFD‐feeding but not in imeglimin‐administered mice (Figure 2e, **left panel**). Genes in Cluster 2 included those involved in lipid metabolism, such as *Elovl2*, *Cideb*, *Slc25a45*, *Cpt1a* and *Plin2*, suggesting an alteration in lipid metabolism in the EDL by imeglimin. The genes in Cluster 7 showed a significant overlap with the top 20 DEG (Figure 2e, **right panel**) and a significant enrichment in inflammatory as well as immediate‐early genes (Figure 2f). Importantly, changes in the TGF‐β signalling, IEG and inflammatory signalling pathways have all been suggested to be associated with muscle atrophy under various conditions [10, 12, 13, 14]. The amelioration of these gene expression changes by imeglimin in the EDL of HFD‐induced obese mice further validated at the gene expression level that imeglimin attenuated HFD‐associated muscle atrophy.

On the other hand, imeglimin‐mediated reduction in inflammatory gene expression in skeletal muscle was not associated with the changes in canonical inflammatory signalling pathways, including JNK, p38 and IκBα, in EDL muscle under our experimental conditions (Figure S1). Additionally, imeglimin treatment did not significantly alter the activation status of mTOR signalling, nor did it markedly affect the expression of the atrogenes *Trim63* and *Fbxo32* in EDL muscle (Figure S2a,b). Furthermore, imeglimin treatment did not markedly alter the expression of major mitochondrial biogenesis‐ or OXPHOS‐related markers, including *Ppargc1* and OXPHOS complex subunits, in EDL muscle from HFD‐fed mice (Figure S2c). Collectively, these findings indicate that imeglimin attenuated HFD‐induced muscle atrophy and suppressed stress‐responsive and inflammatory gene expression without detectable changes in canonical JNK/p38 signalling.

### 3.3 Imeglimin Attenuated Palmitate‐Induced Inflammatory Gene Expression in Cultured C2C12 Myotubes

**FIGURE 3:**
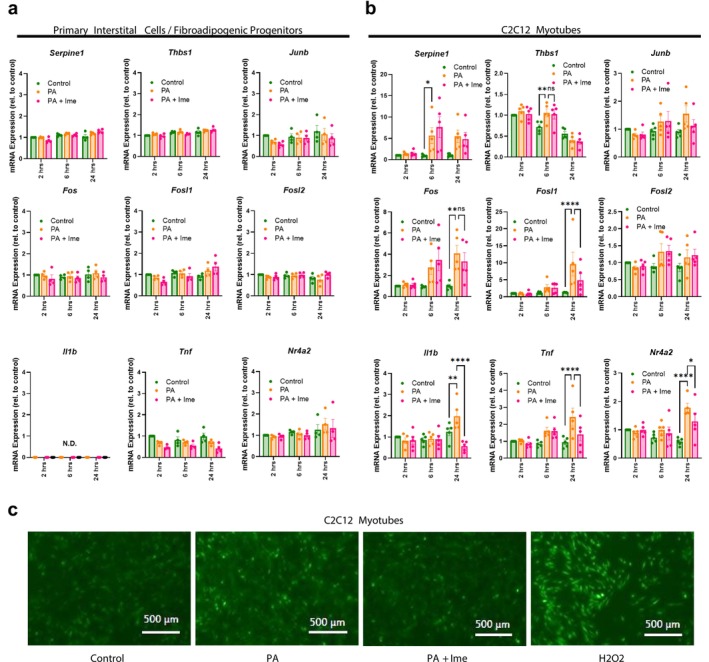
Characterization of fibroadipogenic progenitors and C2C12 myotubes treated with imeglimin under palmitate stimulation. (a, b) Real‐time PCR analyses of indicated gene expressions in (a) primary interstitial cells/fibroadipogenic progenitors isolated from mouse hindlimb (*n* = 4 of four independent mice) or (b) fully differentiated C2C12 myotubes (*n* = 5 of five independent experiments) at indicated time points after palmitate (PA) and imeglimin (Ime) treatments. (c) Representative microscopic images of intracellular ROS levels as detected by DCFH‐DA in fully differentiated C2C12 myotubes after palmitate, imeglimin or H_2_O_2_ treatments. *p < 0.05, **p < 0.01 and ***p < 0.001. The graphs represent mean ± SEM.

The pathology of sarcopenia involves complex intercellular signalling among resident skeletal muscle cells, ultimately leading to global genetic and functional alterations across the entire muscle [10]. Notably, skeletal muscle interstitial cells (SMICs), including fibroadipogenic progenitors (FAPs), play fundamental roles in the characteristic fibro‐fatty infiltration seen in muscle atrophy [15]. To elucidate the mechanism and identify the direct cellular target of imeglimin's anti‐sarcopenic actions under HFD conditions, we employed an in vitro approach and compared the responses of fully differentiated C2C12 myotubes and primary SMICs including FAPs (SMICs/FAPs) to imeglimin under palmitate stimulation. Palmitate stimulation and/or imeglimin treatment of SMICs/FAPs did not alter the expression of profibrotic markers (*Serpine1* and *Thbs1*), IEGs or inflammatory genes (*Junb*, *Fos*, *Il1b* and *Tnf*) at any tested time point (2, 6 and 24 h) (Figure 3a). Conversely, palmitate treatment of fully differentiated C2C12 myotubes resulted in significant induction of *Serpine1* and *Thbs1* at 6 h, followed by upregulation of inflammatory genes, including *Il1b*, *Tnf* and *Nr4a2*, at 24 h (Figure 3b). Co‐stimulation with imeglimin significantly attenuated the palmitate‐induced upregulation of these inflammatory genes (Figure 3b), although it did not affect most IEG expressions, with the exception of *Fosl1*. Collectively, these data suggested that imeglimin's primary anti‐sarcopenic action might involve the direct amelioration of inflammation within the myocytes elicited by lipotoxicity.

Previous reports indicated the essential interactions between sustained inflammation and oxidative stress in the pathologies of muscle atrophy [16], and the specific contribution of palmitate‐induced oxidative stress to the inflammatory responses in myocytes under lipotoxicity [17, 18]. Given the previously suggested imeglimin's cytoprotective effects through ROS reduction [8], we assessed the impact of imeglimin on ROS levels in C2C12 myotubes. Consistent with previous reports, 24 h of palmitate treatment increased ROS levels in fully differentiated C2C12 myotubes as detected by DCFH‐DA dye, whereas imeglimin co‐administration mitigated the ROS induction by palmitate (Figure 3c).

These data indicated that the amelioration of palmitate‐induced inflammation by imeglimin was associated with protection from ROS and sarcopenic gene induction under lipotoxicity and supports the idea that imeglimin may protect against HFD‐associated muscle atrophy primarily through modulation of lipotoxic and inflammatory stress responses in myocytes.

### 3.4 Imeglimin Attenuated Ageing‐Associated Muscle Mass Reduction in Mice

The upregulation of IEGs and inflammatory genes that we observed in the EDL of HFD‐induced obese mice is also a known characteristic of ageing‐associated sarcopenia [10]. Collectively with the potential direct effects of imeglimin on myocytes, we hypothesized that imeglimin could also ameliorate muscle atrophy associated with ageing.

**FIGURE 4:**
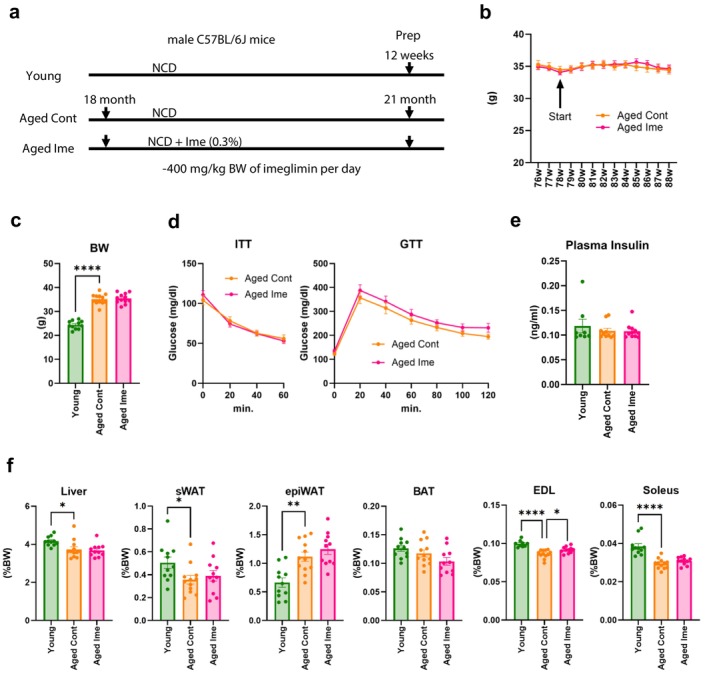
Metabolic parameters and tissue weights in aged mice with imeglimin administration. (a) A scheme showing the experimental setting. (b) Body weight curves, (c) body weights at the end of experiments, (d) average blood glucose concentrations during an insulin tolerance test (ITT) or a glucose tolerance test (GTT) (*n* = 12, except for Young group where *n* = 11). (e) Plasma insulin concentrations of young control mice (Young), control aged mice (Aged Cont) or aged mice fed with NCD + imeglimin (Aged Ime) (*n* = 8, *n* = 10 and *n* = 11, respectively). (f) Tissue weights of liver, subcutaneous white adipose tissue (sWAT), epididymal (eWAT), brown adipose tissue (BAT), EDL and soleus muscles of indicated cohorts of mice (*n* = 12, except for NCD group where *n* = 11). *p < 0.05, **p < 0.01, ***p < 0.001 and **** *p* < 0.0001. For (c) and (f), the statistical significance was evaluated between each cohort and Aged Cont group. For (b) and (d), the statistical significance was evaluated by two‐way ANOVA and post hoc analyses. The graphs represent mean ± SEM.

To test this hypothesis, we next administered imeglimin to male 18‐month‐old aged mice for 12 weeks (Figure 4a). Imeglimin administration did not significantly affect body weights (Figure 4b,c) or glucose metabolism as assessed by insulin and glucose tolerance tests (Figure 4d). Plasma insulin levels were comparable (Figure 4e), whereas the assessment of insulin signalling in the muscles showed that the phosphor‐Akt levels of EDL in imeglimin‐administered aged mice tended to be lower than that of the aged controls (Figure S3). Tissue weight measurements showed differences in those of liver, subcutaneous and epididymal adipose tissue depots between young and aged control mice, whereas imeglimin administration did not affect those tissue weights (Figure 4f, *left panels*). Notably, EDL muscle mass was significantly smaller in the aged control mice compared to young controls, whereas EDL muscle mass was significantly larger in imeglimin‐administered mice than in the aged controls (Figure 4f, *right panels*). These findings suggested that imeglimin had protective effects against muscle atrophy during ageing, which were independent of alterations in systemic glucose homeostasis or direct stimulation of anabolic signalling pathways in skeletal muscle.

### 3.5 Imeglimin Administration in Aged Mice Led to Larger CSA and Attenuated IEG and Inflammatory Gene Induction in EDL

**FIGURE 5:**
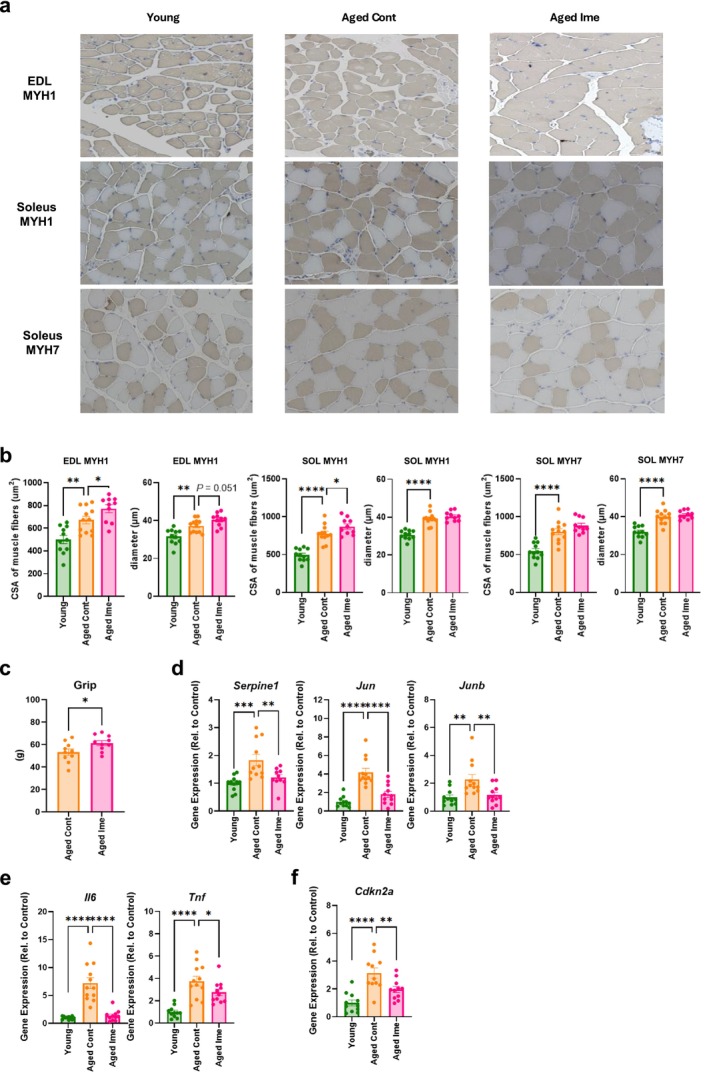
Histology and mRNA expression in skeletal muscle of aged mice with imeglimin administration. (a) The representative histological images of EDL and soleus muscles of young control mice (Young), control aged mice (Aged Cont) or aged mice fed with NCD + imeglimin (Aged Ime) (*n* = 12, except for Young group where *n* = 11). (b) The quantifications of cross‐sectional area (CSA) and diameter of MYH1 (Type IIX/D fast muscle fibres) or MYH7 (Type I slow‐twitch muscle fibres) in EDL or soleus (SOL) (*n* = 11 in Young group, *n* = 12 in Aged Cont group and *n* = 10 in Aged Ime group). (c) Grip strength in control aged mice (Aged Cont) and aged mice treated with imeglimin (Aged Ime) after 7 weeks of treatment (*n* = 9 per group). (d–f) Real‐time PCR analyses of indicated gene expressions in EDL of mice (*n* = 12, except for Young group where *n* = 11). For all of the ANOVA analyses, the statistical significance was evaluated between each cohort and Aged Cont group. *p < 0.05, **p < 0.01, *** *p* < 0.001 and ****p < 0.0001. The graphs represent mean ± SEM.

Histological assessments revealed that both the CSA and diameter of Type IIX/D fast‐twitch fibres (stained with MYH1) in the EDL of aged mice administered with imeglimin were significantly larger compared to aged controls (Figure 5a,b). The CSA and diameter of Type I slow‐twitch fibres (stained with MYH7) in the soleus muscle were comparable between the two aged mouse cohorts, whereas the CSA of Type IIX/D fibres in the soleus were also significantly larger in the imeglimin‐administered group compared to aged controls. Paradoxically, the CSA of these respective muscle fibres was larger in the aged control mice compared to young control mice, which may be due to differences in their developmental stages. Moreover, the dissociation between reduced muscle mass and increased myofibre CSA in aged EDL may indicate age‐associated remodelling characterized by selective myofibre loss and compensatory enlargement of surviving fibres. To further evaluate whether the morphological improvements in skeletal muscle were accompanied by functional benefits, we measured grip strength in an independent cohort of aged mice after 7 weeks of imeglimin treatment. Imeglimin‐treated aged mice exhibited significantly greater grip strength compared with age‐matched control mice (Figure 5c), supporting the possibility that imeglimin ameliorated not only morphological but also functional decline of fast‐twitch muscles during ageing. Gene expression analyses of EDL showed that *Serpine1*, *Jun* and *Junb*, as well as typical inflammatory genes, were more highly expressed in aged control mice compared to young mice. Importantly, these expressions were lower in the imeglimin‐administered mice compared to aged controls (Figure 5d,e). These data collectively indicated that imeglimin administration counteracted ageing‐induced changes in the EDL, in a similar manner as observed under the HFD‐induced obese condition. Interestingly, the EDL expression of *Cdkn2a*, an established marker of senescence, was higher in aged control mice than in young mice but was significantly lower in the imeglimin‐administered aged mice (Figure 5f). This suggests that imeglimin may have anti‐senescence effects in EDL.

Skeletal muscles and bones are closely linked, and our previous study indicated that protection from sarcopenia could lead to amelioration of osteoporosis [19]. Interestingly, exploratory micro‐CT analyses showed partial improvement in BMD, BS/BV and decreased plasma CTX‐1 in aged mice treated with imeglimin (Figure S4a–f), suggesting that imeglimin protected mice from osteopenia by inhibiting bone resorption.

## 4 Discussion

This study demonstrates that imeglimin, a novel antidiabetic agent, possesses unique pharmacological properties in skeletal muscle that extend beyond glycaemic control. Specifically, imeglimin effectively prevented skeletal muscle atrophy in both HFD‐induced obesity and naturally aged mouse models. Notably, the protective effects were observed predominantly in the EDL muscle, which is rich in Type IIX/D fast‐twitch fibres, with minimal effect on the soleus muscle, composed primarily of Type IIA fast‐twitch fibres and Type I slow‐twitch fibres. This is a significant finding, as Type IIX/D fibres are particularly susceptible to degeneration in ageing and metabolic disorders [20], and their atrophy is a hallmark of sarcopenia. Imeglimin not only preserved muscle mass but also counteracted functional decline in aged skeletal muscle, demonstrating benefits beyond morphology. In line with our findings, a recent clinical study reported that short‐term imeglimin treatment improved muscle strength in patients with Type 2 diabetes [21]. Our study adds mechanistic insight to this observation, demonstrating improvements in gene expression profiles, relative muscle mass and histological architecture under pathological conditions.

At the molecular level, imeglimin treatment reversed the upregulation of several genes associated with HFD‐associated or age‐related muscle atrophy, including immediate early genes and key components of the TGF‐β and TNF inflammatory pathways, the well‐established drivers of muscle protein degradation and impaired synthesis [22, 23, 24]. Among these, although Serpine1/PAI‐1 expression was markedly reduced in EDL muscle in vivo, imeglimin did not directly suppress *Serpine1* expression in isolated SMICs or C2C12 myotubes under palmitate stimulation. These findings suggest that the reduction of PAI‐1 observed at the tissue level may not primarily reflect a direct effect on FAPs themselves, but rather secondary remodelling of the pathological muscle microenvironment. Given the complex intercellular crosstalk among myofibres, interstitial cells, immune cells and extracellular matrix components in skeletal muscle, imeglimin‐mediated attenuation of myocyte stress responses may indirectly reshape fibro‐inflammatory signalling within the muscles.

**FIGURE 6:**
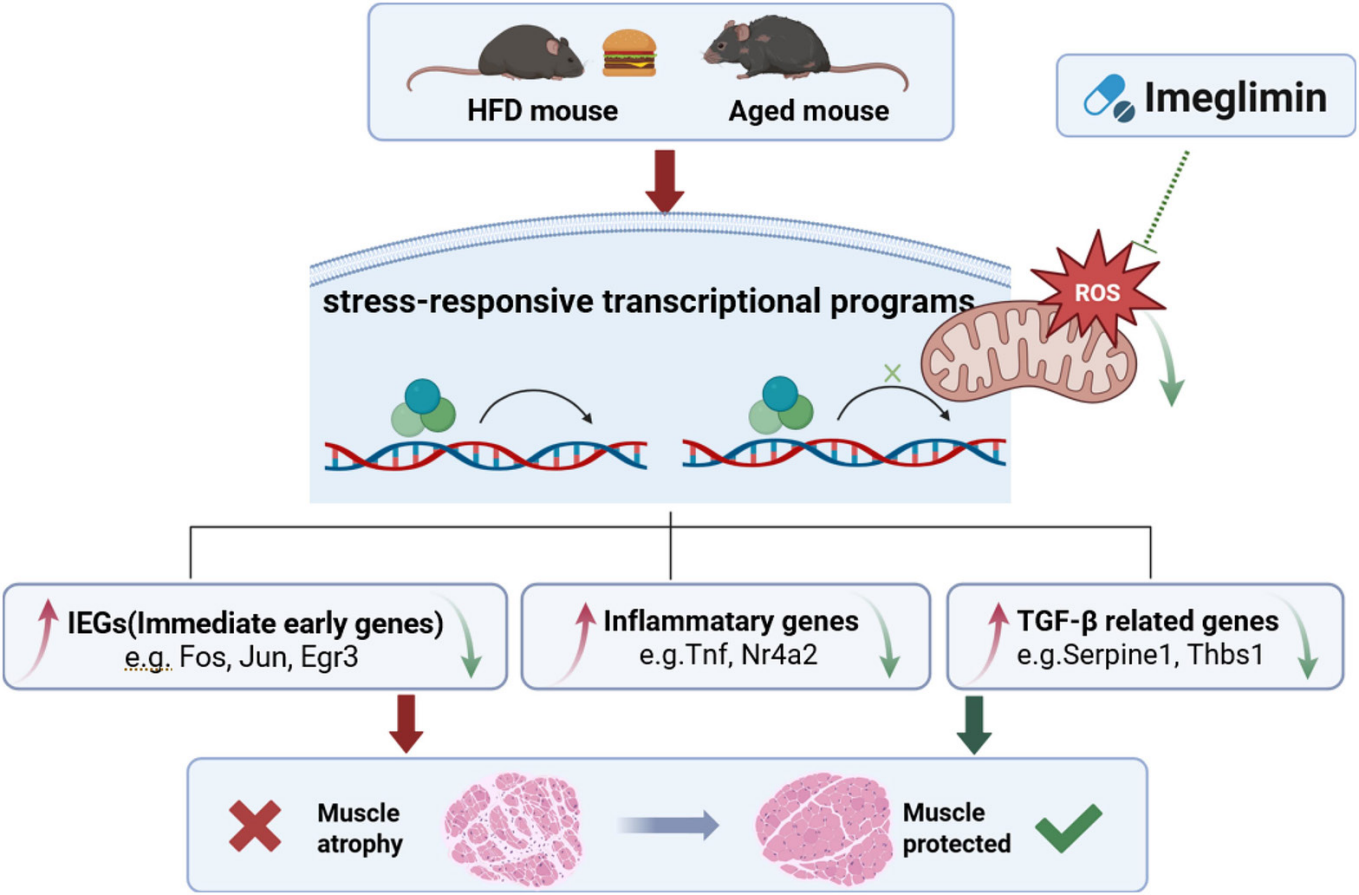
Schematic diagram of the protective mechanism of imeglimin against obesity‐ and ageing‐associated muscle atrophy.

The precise mechanism underlying the action of imeglimin, however, remains to be fully elucidated. Interestingly, in nondiabetic aged mice, imeglimin did not significantly alter systemic glucose tolerance, suggesting that their muscle‐protective effects may occur independently of their glucose‐lowering actions. Instead, these effects appear to involve direct regulation of intrinsic gene expression and metabolic programmes within skeletal muscle. An important finding of this study is that imeglimin‐mediated reduction in inflammatory gene expression in skeletal muscle was not associated with the typical inflammatory or stress‐related signalling pathways including JNK, p38, IκBα and MAPK. Furthermore, imeglimin consistently reduced palmitate‐induced inflammatory responses and ROS accumulation in C2C12 myotubes under our experimental conditions, suggesting the possibility that myocytes themselves represent a direct cellular target of imeglimin under chronic stress conditions. These findings indicate that the observed anti‐inflammatory transcriptional changes in vivo may reflect redox‐dependent transcriptional reprogramming rather than acute suppression of upstream inflammatory kinase cascades. Emerging evidence highlights imeglimin's role in improving mitochondrial function by rebalancing respiratory chain activity [25, 26]. Mitochondrial ROS is increasingly recognized as a regulator of inflammatory transcriptional programmes independently of classical MAPK or NF‐κB activation states [27]. Therefore, reduced mitochondrial oxidative stress may contribute to the attenuation of inflammatory and fibro‐inflammatory gene expression observed in the present study. From this perspective, the suppression of inflammatory, immediate early genes and TGF‐β‐associated genes observed in the present study may reflect attenuation of chronic redox‐dependent stress responses within metabolically challenged myofibres. Notably, despite the transcriptional improvement in inflammatory and stress‐responsive pathways, imeglimin did not significantly alter the expression of the atrogenes Trim63 or Fbxo32 in EDL muscle in vivo. This result may reflect that protective effects of imeglimin may primarily involve attenuation of lipotoxic and inflammatory stress responses rather than direct suppression of the FoxO‐atrogene axis. The schematic diagram of the potential mechanism of imeglimin against obesity and age‐related muscle atrophy is shown in Figure 6.

The preferential effects of imeglimin in EDL muscle may also be mechanistically relevant. Fast‐twitch glycolytic fibres generally possess lower oxidative buffering capacity and exhibit greater vulnerability to lipid overload, mitochondrial stress and inflammatory injury than oxidative slow‐twitch fibres. Such intrinsic metabolic characteristics may render fast‐twitch fibres more responsive to interventions targeting lipotoxic or oxidative stress pathways [28]. Consistent with this interpretation, differentiated C2C12 myotubes, which expressed MYH1/Type IIX/D fibres associated markers, showed robust responsiveness to both palmitate and imeglimin in our in vitro experiments. Obesity and ageing share multiple pathogenic mechanisms, including chronic inflammation, lipotoxicity, oxidative stress and impaired metabolic adaptation within skeletal muscle. Although the precise basis for the fibre type‐selective effects remained unresolved, our findings raise the possibility that imeglimin preferentially protected metabolically vulnerable myofibres under conditions of obesity and ageing.

Current antidiabetic therapies have not been established to protect against sarcopenia. Metformin, the precursor drug of imeglimin, has shown inconsistent effects on muscle function, with some studies reporting benefits [29, 30, 31] and others suggesting limited efficacy or poor tolerability in frail older individuals [32]. In contrast, glucagon‐like peptide (GLP)‐1 receptor agonists, GLP‐1/glucose‐dependent insulinotropic polypeptide (GIP) receptor dual agonist and sodium‐glucose cotransporter (SGLT)‐2 inhibitors are frequently associated with loss of lean mass during weight reduction, potentially increasing the risk of sarcopenia [33, 34, 35, 36, 37]. In this context, imeglimin exhibited a distinct profile characterized by muscle preservation together with glycaemic and metabolic benefits. Importantly, imeglimin has demonstrated favourable safety and tolerability in multiple clinical studies, including in combination with metformin or SGLT‐2 inhibitors [6, 38]. Exploratory analyses also suggested beneficial effects of imeglimin on bone‐related parameters and suppression of ageing‐associated bone resorption, potentially through modulation of mitochondrial function in osteoclasts [39]. Collectively, these findings raise the possibility that imeglimin may possess a uniquely favourable musculoskeletal profile among current antidiabetic agents and may represent a metabolically targeted strategy for preserving musculoskeletal health in ageing and obesity‐associated diabetes.

Several limitations should be acknowledged. First, transcriptomic analyses were performed using bulk RNA sequencing and therefore could not resolve fibre‐type‐specific or cell‐type‐specific transcriptional responses within skeletal muscle. Future studies using single‐nucleus transcriptomics or single‐fibre approaches will be important to dissect the cellular heterogeneity underlying the observed phenotypes. Second, although grip strength was improved in aged mice, comprehensive functional assessments such as endurance capacity, coordination or fatigue resistance were not evaluated. Third, the present study was conducted exclusively in male mice, and potential sex‐dependent effects require further investigation.

In conclusion, this study identifies imeglimin as a modulator of skeletal muscle atrophy in obesity and ageing, accompanied by suppression of stress‐ and inflammation‐associated transcriptional programmes rather than broad activation of anabolic or mitochondrial pathways. These findings support the concept that targeting redox‐sensitive metabolic stress responses may represent a therapeutically relevant strategy for preserving skeletal muscle homeostasis under metabolic and ageing‐related stress.

## Funding

This work was supported by Sumitomo Pharma. Sumitomo Pharma was not involved in the design of the study; the collection, analysis and interpretation of data; writing the report; and did not impose any restrictions regarding the publication of the report.

## Conflicts of Interest

The authors declare no conflicts of interest.

## Supporting information


**Data S1:** Supporting information.

**Figure S1:** Inflammatory and stress‐responsive signalling pathways in the EDL muscle (related to Figure 2). Representative western blot images of p‐JNK, JNK, p‐p38, p38, p‐IκBα and IκBα in EDL muscle from mice fed with normal chow diet (NCD), high‐fat diet (HFD) or HFD with imeglimin administration (HFD Ime). *p < 0.05, **p < 0.01 and ***p < 0.001. Data are presented as mean ± SEM.
**Figure S2:** Canonical metabolic, atrophy and mitochondrial signalling pathways in the EDL muscles (related to Figure 3). (a) Representative western blot images of p‐mTOR and mTOR in EDL muscle from NCD, HFD and HFD Ime mice. (b) Real‐time PCR analyses of atrophy‐related genes Trim63 and Fbxo32 in EDL muscle from NCD, HFD and HFD Ime mice (*n* = 10). (c) Real‐time PCR analyses of mitochondrial biogenesis‐ and OXPHOS‐related genes, including Timm8b, *Uqcrh*, *Rnr2*, *Ndufv2*, Cox6c and Ppargc1, in EDL muscle from mice fed with normal chow diet (NCD), high‐fat diet (HFD) or HFD with imeglimin administration (HFD Ime) (*n* = 10, left panel). Representative western blot images of Cox IV and OXPHOS complex subunits (CI–CV) in EDL muscle from NCD, HFD and HFD Ime mice (right panel). Calnexin was used as a loading control. *p < 0.05, **p < 0.01 and ***p < 0.001. Data are presented as mean ± SEM.
**Figure S3:** AKT signalling in EDL muscle from aged mice with imeglimin administration (related to Figure 4). Representative western blot images and quantification analyses of p‐AKT and AKT in skeletal muscle from young control mice (Young), control aged mice (Aged Cont) or aged mice fed with NCD + imeglimin (Aged Ime) (*n* = 6). Data are presented as mean ± SEM.
**Figure S4:** Bone morphology of aged mice with imeglimin administration. (a) Representative images by micro‐CT scanning of femur from young control mice (Young), control aged mice (Aged Cont) or aged mice fed with NCD + imeglimin (Aged Ime). (b) Quantifications of bone volume (BV) of total, cortical and cancellous bone of femur (*n* = 12, except for Young group where *n* = 11). (c) Quantifications of bone volume (BV)/total tissue volume (TV) of total and cancellous bone of femur (*n* = 12, except for Young group where *n* = 11). (d) Bone mineral density (BMD) of total, cortical and cancellous bone of femur (*n* = 12, except for Young group where *n* = 11). (e) Quantifications of bone surface (BS)/bone volume (BV) of total, cortical and cancellous bone of femur (*n* = 12, except for Young group where *n* = 11). (f) Plasma concentrations of bone turnover markers (*n* = 11, except for Aged Cont where *n* = 12 and Aged Ime where *n* = 10). *p < 0.05, **p < 0.01, ***p < 0.001 and ****p < 0.0001. Data are presented as mean ± SEM.

## Data Availability

The raw RNA sequencing data generated in this study have been deposited in the GEO database under the accession number GSE334796. All raw data included in this study will be made available upon request.
