# Supplementary figures and images for: Imeglimin Attenuates Skeletal Muscle Atrophy in Mouse Models of Obesity and Ageing
Source: J Cachexia Sarcopenia Muscle. 2026 Jul 28;17(4):e70354. doi: 10.1002/jcsm.70354 (PMC13416397; doi:10.1002/jcsm.70354)

# Supplementary Fig 1

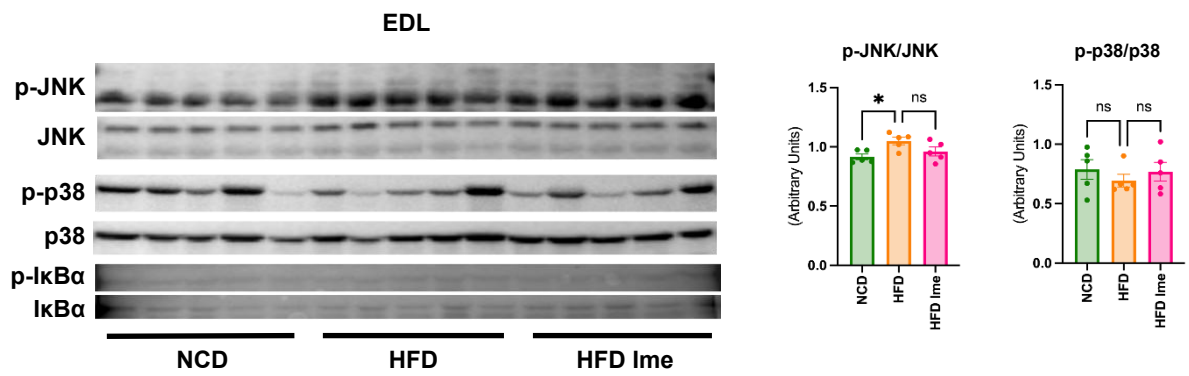

# Supplementary Fig 2

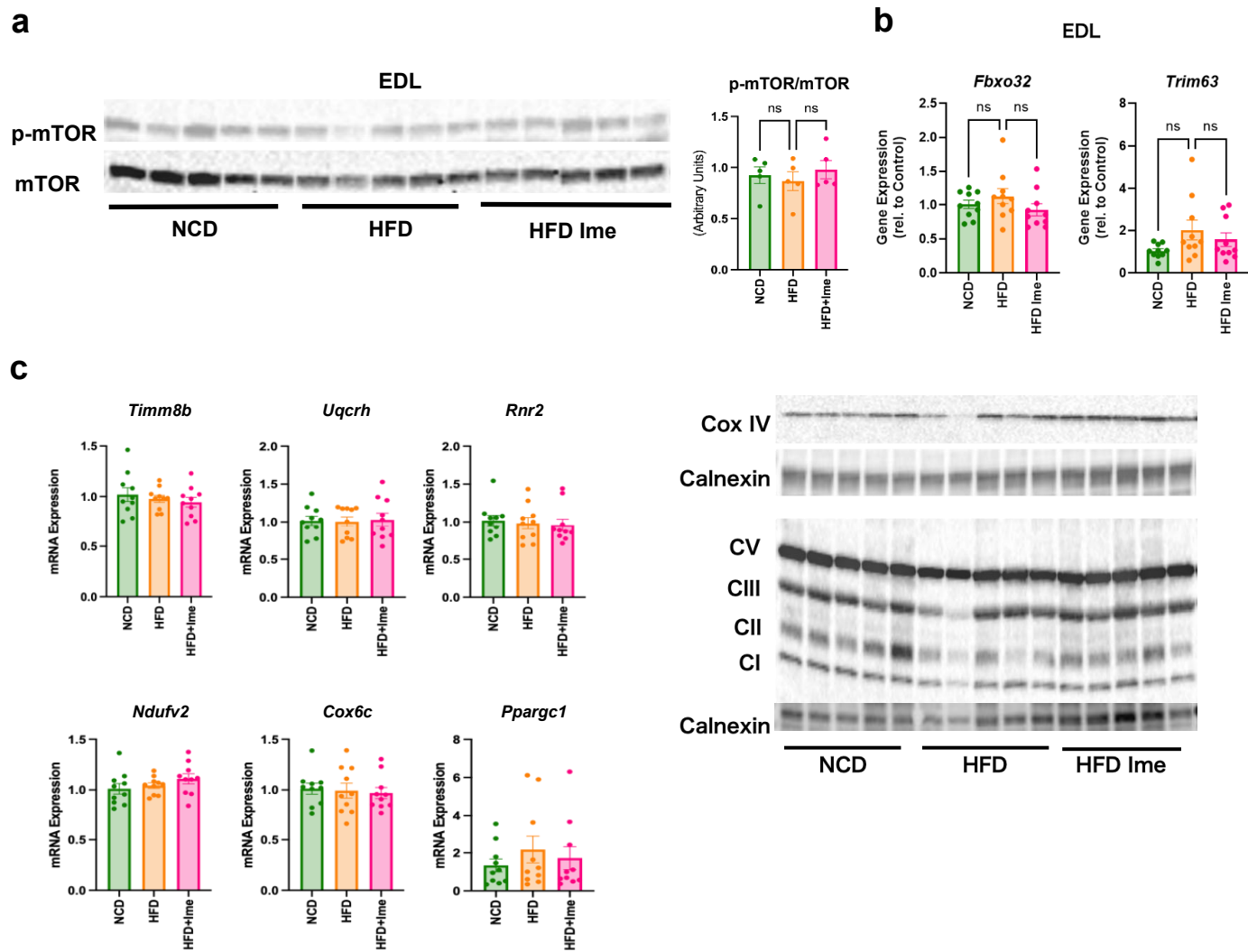

# Supplementary Fig 3

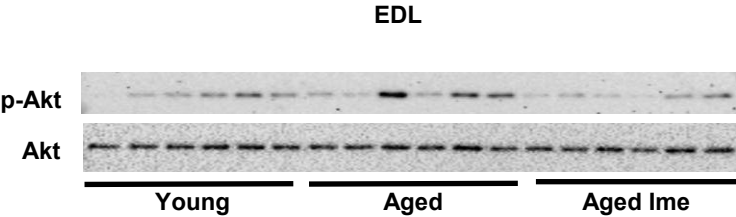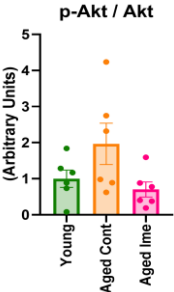

# Supplementary Fig 4

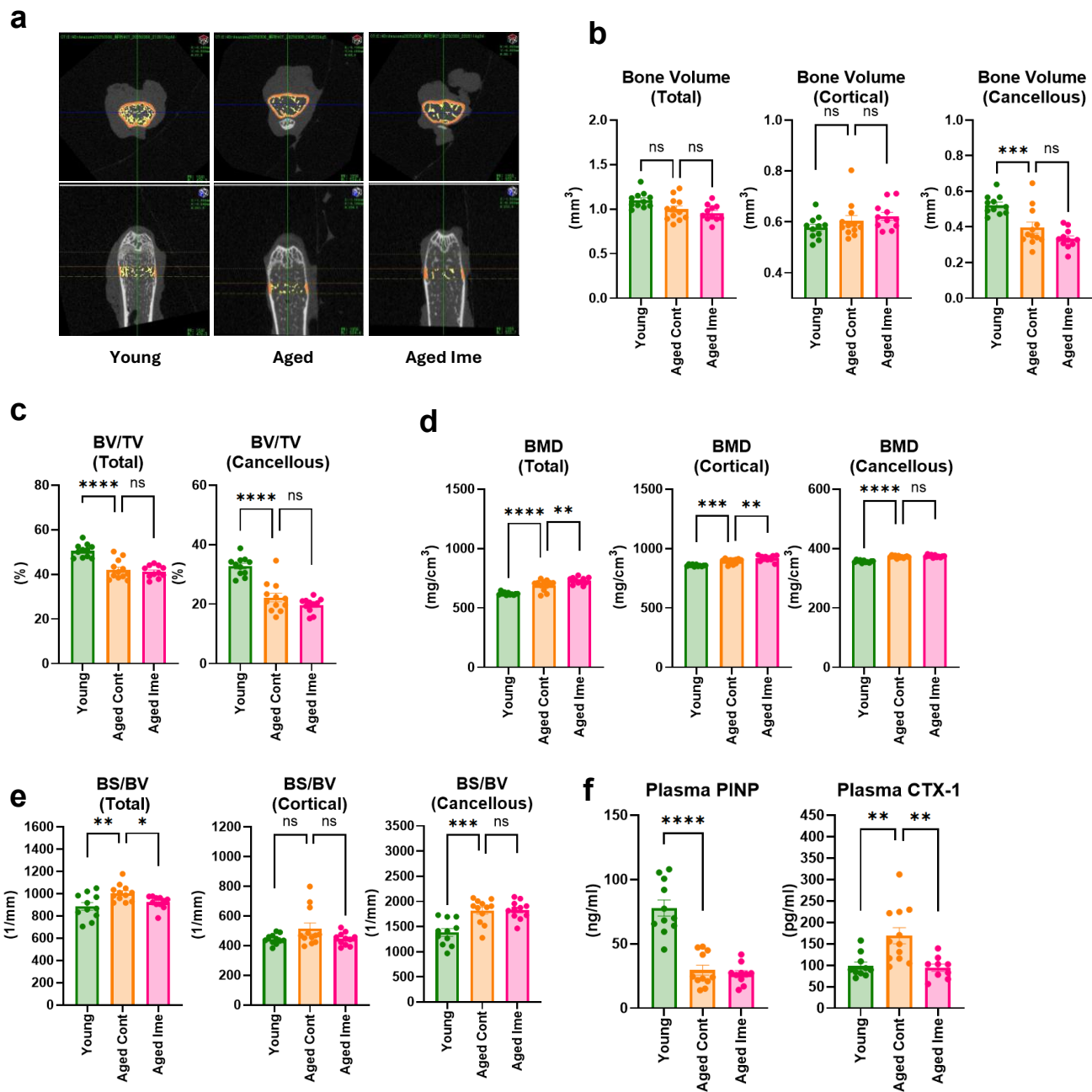

Supplement: Supplementary file 1 — Data S1: Supporting information. [file JCSM-17-e70354-s002.pdf]
