# Supplementary material for: Imeglimin Attenuates Skeletal Muscle Atrophy in Mouse Models of Obesity and Ageing
Source: J Cachexia Sarcopenia Muscle. 2026 Jul 28;17(4):e70354. doi: 10.1002/jcsm.70354 (PMC13416397; doi:10.1002/jcsm.70354)
Supplement: Supplementary file 2 — Figure S1: Inflammatory and stress‐responsive signalling pathways in the EDL muscle (related to Figure 2). Representative western blot images of p‐JNK, JNK, p‐p38, p38, p‐IκBα and IκBα in EDL muscle from mice fed with normal chow diet (NCD), high‐fat diet (HFD) or HFD with imeglimin administration (HFD Ime). *p < 0.05, **p < 0.01 and ***p < 0.001. Data are presented as mean ± SEM. Figure S2: Canonical metabolic, atrophy and mitochondrial signalling pathways in the EDL muscles (related to Figure 3). (a) Representative western blot images of p‐mTOR and mTOR in EDL muscle from NCD, HFD and HFD Ime mice. (b) Real‐time PCR analyses of atrophy‐related genes Trim63 and Fbxo32 in EDL muscle from NCD, HFD and HFD Ime mice (n = 10). (c) Real‐time PCR analyses of mitochondrial biogenesis‐ and OXPHOS‐related genes, including Timm8b, Uqcrh, Rnr2, Ndufv2, Cox6c and Ppargc1, in EDL muscle from mice fed with normal chow diet (NCD), high‐fat diet (HFD) or HFD with imeglimin administration (HFD Ime) (n = 10, left panel). Representative western blot images of Cox IV and OXPHOS complex subunits (CI–CV) in EDL muscle from NCD, HFD and HFD Ime mice (right panel). Calnexin was used as a loading control. *p < 0.05, **p < 0.01 and ***p < 0.001. Data are presented as mean ± SEM. Figure S3: AKT signalling in EDL muscle from aged mice with imeglimin administration (related to Figure 4). Representative western blot images and quantification analyses of p‐AKT and AKT in skeletal muscle from young control mice (Young), control aged mice (Aged Cont) or aged mice fed with NCD + imeglimin (Aged Ime) (n = 6). Data are presented as mean ± SEM. Figure S4: Bone morphology of aged mice with imeglimin administration. (a) Representative images by micro‐CT scanning of femur from young control mice (Young), control aged mice (Aged Cont) or aged mice fed with NCD + imeglimin (Aged Ime). (b) Quantifications of bone volume (BV) of total, cortical and cancellous bone of femur (n = 12, except for Young group wher [file JCSM-17-e70354-s001.docx]

**Supplementary Fig. 1 Inflammatory and Stress-responsive Signaling Pathways in the EDL Muscle (Related to Fig. 2)**

Representative western blot images of p-JNK, JNK, p-p38, p38, p-IκBα, and IκBα in EDL muscle from mice fed with normal chow diet (NCD), high-fat diet (HFD), or HFD with imeglimin administration (HFD Ime). **P* < 0.05, ***P* < 0.01 and ****P* < 0.001. Data are presented as mean ± s.e.m.

**Supplementary Fig. 2 Canonical Metabolic, Atrophy, and Mitochondrial Signaling Pathways in the EDL Muscles (Related to Fig. 3)**

1. Representative western blot images of p-mTOR and mTOR in EDL muscle from NCD, HFD, and HFD Ime mice. **(b)** Real-time PCR analyses of atrophy-related genes Trim63 and Fbxo32 in EDL muscle from NCD, HFD, and HFD Ime mice (*n* = 10). **(c)** Real-time PCR analyses of mitochondrial biogenesis- and OXPHOS-related genes, including Timm8b, *Uqcrh*, *Rnr2*, *Ndufv2*, Cox6c, and Ppargc1, in EDL muscle from mice fed with normal chow diet (NCD), high-fat diet (HFD), or HFD with imeglimin administration (HFD Ime) (n = 10, Left panel). Representative western blot images of Cox IV and OXPHOS complex subunits (CI–CV) in EDL muscle from NCD, HFD, and HFD Ime mice (Right panel). Calnexin was used as a loading control. **P* < 0.05, ***P* < 0.01 and ****P* < 0.001. Data are presented as mean ± s.e.m.

**Supplementary Fig. 3 AKT Signaling in EDL Muscle from Aged Mice with Imeglimin Administration (Related to Fig. 4)**

Representative western blot images and quantification analyses of p-AKT and AKT in skeletal muscle from young control mice (Young), control aged mice (Aged Cont), or aged mice fed with NCD + imeglimin (Aged Ime) (*n* = 6). Data are presented as mean ± s.e.m.

**Supplementary Fig. 4 Bone Morphology of Aged Mice with Imeglimin Administration**

**(a)** Representative images by micro-CT scanning of femur from young control mice (Young), control aged mice (Aged Cont) or aged mice fed with NCD + imeglimin (Aged Ime). **(b)** Quantifications of bone volume (BV) of total, cortical and cancellous bone of femur (*n* = 12, except for Young group where *n* = 11). **(c)** Quantifications of bone volume (BV) / total tissue volume (TV) of total and cancellous bone of femur (*n* = 12, except for Young group where *n* = 11). **(d)** Bone mineral density (BMD) of total, cortical and cancellous bone of femur (*n* = 12, except for Young group where *n* = 11). **(e)** Quantifications of bone surface (BS) / bone volume (BV) of total, cortical and cancellous bone of femur (*n* = 12, except for Young group where *n* = 11). **(f)** Plasma concentrations of bone turnover markers (*n* = 11, except for Aged Cont where *n* = 12 and Aged Ime where *n* = 10). **P* < 0.05, ***P* < 0.01, ****P* < 0.001 and *****P* < 0.0001. Data are presented as mean ± s.e.m.
